# Supplementary material for: Resolving the full spectrum of human genome variation using Linked-Reads
Source: Genome Res. 2019 Apr;29(4):635–45. doi: 10.1101/gr.234443.118 (PMC6442396; doi:10.1101/gr.234443.118)
Supplement: Supplemental Material [file supp_gr.234443.118_Supplemental_longranger-2.2.zip › longranger-2.2.tar.bz2!/lr2.2-public/lib/stan/cmdstan/stan/doc/api/html/agrad_2rev_2matrix_2mdivide__left__ldlt_8hpp_source.html]

Stan: src/stan/agrad/rev/matrix/mdivide\_left\_ldlt.hpp Source File


|  |  |
| --- | --- |
|  | Stan  2.5.0  probability, sampling & optimization |


- Main Page
- Related Pages
- Namespaces
- Classes
- Files

- File List
- File Members

All Classes Namespaces Files Functions Variables Typedefs Enumerations Enumerator Friends Macros Pages

- src
- stan
- agrad
- rev
- matrix

mdivide\_left\_ldlt.hpp

Go to the documentation of this file.

1 #ifndef STAN\_\_AGRAD\_\_REV\_\_MATRIX\_\_MDIVIDE\_LEFT\_LDLT\_HPP

2 #define STAN\_\_AGRAD\_\_REV\_\_MATRIX\_\_MDIVIDE\_LEFT\_LDLT\_HPP

3

4 #include <stan/math/matrix/Eigen.hpp>

5 #include <stan/agrad/rev/var.hpp>

6 #include <stan/agrad/rev/matrix/LDLT\_alloc.hpp>

7 #include <stan/agrad/rev/matrix/LDLT\_factor.hpp>

8 #include <stan/math/error\_handling/matrix/check\_multiplicable.hpp>

9

10 namespace stan {

11  namespace agrad {

12  namespace {

13  template <int R1,int C1,int R2,int C2>

14  class mdivide\_left\_ldlt\_alloc : public chainable\_alloc {

15  public:

16  virtual ~mdivide\_left\_ldlt\_alloc() {}

17

23  boost::shared\_ptr< Eigen::LDLT< Eigen::Matrix<double,R1,C1> > > \_ldltP;

24  Eigen::Matrix<double,R2,C2> C\_;

25  };

26

37  template <int R1,int C1,int R2,int C2>

38  class mdivide\_left\_ldlt\_vv\_vari : public vari {

39  public:

40  int M\_; // A.rows() = A.cols() = B.rows()

41  int N\_; // B.cols()

42  vari\*\* \_variRefB;

43  vari\*\* \_variRefC;

44  mdivide\_left\_ldlt\_alloc<R1,C1,R2,C2> \*\_alloc;

45  const LDLT\_alloc<R1,C1> \*\_alloc\_ldlt;

46

47  mdivide\_left\_ldlt\_vv\_vari(const stan::math::LDLT\_factor<var,R1,C1> &A,

48  const Eigen::Matrix<var,R2,C2> &B)

49  : vari(0.0),

50  M\_(A.rows()),

51  N\_(B.cols()),

52  \_variRefB((vari\*\*)stan::agrad::memalloc\_.alloc(sizeof(vari\*)

53  \* B.rows() \* B.cols())),

54  \_variRefC((vari\*\*)stan::agrad::memalloc\_.alloc(sizeof(vari\*)

55  \* B.rows() \* B.cols())),

56  \_alloc(new mdivide\_left\_ldlt\_alloc<R1,C1,R2,C2>()),

57  \_alloc\_ldlt(A.\_alloc)

58  {

59  int pos = 0;

60  \_alloc->C\_.resize(M\_,N\_);

61  for (int j = 0; j < N\_; j++) {

62  for (int i = 0; i < M\_; i++) {

63  \_variRefB[pos] = B(i,j).vi\_;

64  \_alloc->C\_(i,j) = B(i,j).val();

65  pos++;

66  }

67  }

68

69  \_alloc\_ldlt->\_ldlt.solveInPlace(\_alloc->C\_);

70

71  pos = 0;

72  for (int j = 0; j < N\_; j++) {

73  for (int i = 0; i < M\_; i++) {

74  \_variRefC[pos] = new vari(\_alloc->C\_(i,j),false);

75  pos++;

76  }

77  }

78  }

79

80  virtual void chain() {

81  Eigen::Matrix<double,R1,C1> adjA(M\_,M\_);

82  Eigen::Matrix<double,R2,C2> adjB(M\_,N\_);

83

84  int pos = 0;

85  for (int j = 0; j < N\_; j++)

86  for (int i = 0; i < M\_; i++)

87  adjB(i,j) = \_variRefC[pos++]->adj\_;

88

89  \_alloc\_ldlt->\_ldlt.solveInPlace(adjB);

90  adjA.noalias() = -adjB \* \_alloc->C\_.transpose();

91

92  for (int j = 0; j < M\_; j++)

93  for (int i = 0; i < M\_; i++)

94  \_alloc\_ldlt->\_variA(i,j)->adj\_ += adjA(i,j);

95

96  pos = 0;

97  for (int j = 0; j < N\_; j++)

98  for (int i = 0; i < M\_; i++)

99  \_variRefB[pos++]->adj\_ += adjB(i,j);

100  }

101  };

102

113  template <int R1,int C1,int R2,int C2>

114  class mdivide\_left\_ldlt\_dv\_vari : public vari {

115  public:

116  int M\_; // A.rows() = A.cols() = B.rows()

117  int N\_; // B.cols()

118  vari\*\* \_variRefB;

119  vari\*\* \_variRefC;

120  mdivide\_left\_ldlt\_alloc<R1,C1,R2,C2> \*\_alloc;

121

122  mdivide\_left\_ldlt\_dv\_vari(const stan::math::LDLT\_factor<double,R1,C1> &A,

123  const Eigen::Matrix<var,R2,C2> &B)

124  : vari(0.0),

125  M\_(A.rows()),

126  N\_(B.cols()),

127  \_variRefB((vari\*\*)stan::agrad::memalloc\_.alloc(sizeof(vari\*)

128  \* B.rows() \* B.cols())),

129  \_variRefC((vari\*\*)stan::agrad::memalloc\_.alloc(sizeof(vari\*)

130  \* B.rows() \* B.cols())),

131  \_alloc(new mdivide\_left\_ldlt\_alloc<R1,C1,R2,C2>())

132  {

133  using Eigen::Matrix;

134  using Eigen::Map;

135

136  int pos = 0;

137  \_alloc->C\_.resize(M\_,N\_);

138  for (int j = 0; j < N\_; j++) {

139  for (int i = 0; i < M\_; i++) {

140  \_variRefB[pos] = B(i,j).vi\_;

141  \_alloc->C\_(i,j) = B(i,j).val();

142  pos++;

143  }

144  }

145

146  \_alloc->\_ldltP = A.\_ldltP;

147  \_alloc->\_ldltP->solveInPlace(\_alloc->C\_);

148

149  pos = 0;

150  for (int j = 0; j < N\_; j++) {

151  for (int i = 0; i < M\_; i++) {

152  \_variRefC[pos] = new vari(\_alloc->C\_(i,j),false);

153  pos++;

154  }

155  }

156  }

157

158  virtual void chain() {

159  Eigen::Matrix<double,R2,C2> adjB(M\_,N\_);

160

161  int pos = 0;

162  for (int j = 0; j < adjB.cols(); j++)

163  for (int i = 0; i < adjB.rows(); i++)

164  adjB(i,j) = \_variRefC[pos++]->adj\_;

165

166  \_alloc->\_ldltP->solveInPlace(adjB);

167

168  pos = 0;

169  for (int j = 0; j < adjB.cols(); j++)

170  for (int i = 0; i < adjB.rows(); i++)

171  \_variRefB[pos++]->adj\_ += adjB(i,j);

172  }

173  };

174

185  template <int R1,int C1,int R2,int C2>

186  class mdivide\_left\_ldlt\_vd\_vari : public vari {

187  public:

188  int M\_; // A.rows() = A.cols() = B.rows()

189  int N\_; // B.cols()

190  vari\*\* \_variRefC;

191  mdivide\_left\_ldlt\_alloc<R1,C1,R2,C2> \*\_alloc;

192  const LDLT\_alloc<R1,C1> \*\_alloc\_ldlt;

193

194  mdivide\_left\_ldlt\_vd\_vari(const stan::math::LDLT\_factor<var,R1,C1> &A,

195  const Eigen::Matrix<double,R2,C2> &B)

196  : vari(0.0),

197  M\_(A.rows()),

198  N\_(B.cols()),

199  \_variRefC((vari\*\*)stan::agrad::memalloc\_.alloc(sizeof(vari\*)

200  \* B.rows() \* B.cols())),

201  \_alloc(new mdivide\_left\_ldlt\_alloc<R1,C1,R2,C2>()),

202  \_alloc\_ldlt(A.\_alloc)

203  {

204  \_alloc->C\_ = B;

205  \_alloc\_ldlt->\_ldlt.solveInPlace(\_alloc->C\_);

206

207  int pos = 0;

208  for (int j = 0; j < N\_; j++) {

209  for (int i = 0; i < M\_; i++) {

210  \_variRefC[pos] = new vari(\_alloc->C\_(i,j),false);

211  pos++;

212  }

213  }

214  }

215

216  virtual void chain() {

217  Eigen::Matrix<double,R1,C1> adjA(M\_,M\_);

218  Eigen::Matrix<double,R1,C2> adjC(M\_,N\_);

219

220  int pos = 0;

221  for (int j = 0; j < adjC.cols(); j++)

222  for (int i = 0; i < adjC.rows(); i++)

223  adjC(i,j) = \_variRefC[pos++]->adj\_;

224

225  adjA = -\_alloc\_ldlt->\_ldlt.solve(adjC\*\_alloc->C\_.transpose());

226

227  for (int j = 0; j < adjA.cols(); j++)

228  for (int i = 0; i < adjA.rows(); i++)

229  \_alloc\_ldlt->\_variA(i,j)->adj\_ += adjA(i,j);

230  }

231  };

232  }

233

241  template <int R1,int C1,int R2,int C2>

242  inline Eigen::Matrix<var,R1,C2>

243  mdivide\_left\_ldlt(const stan::math::LDLT\_factor<var,R1,C1> &A,

244  const Eigen::Matrix<var,R2,C2> &b) {

245  Eigen::Matrix<var,R1,C2> res(b.rows(),b.cols());

246

247  stan::math::check\_multiplicable("mdivide\_left\_ldlt(%1%)",A,"A",

248  b,"b",(double\*)0);

249

250  mdivide\_left\_ldlt\_vv\_vari<R1,C1,R2,C2> \*baseVari = new mdivide\_left\_ldlt\_vv\_vari<R1,C1,R2,C2>(A,b);

251

252  int pos = 0;

253  for (int j = 0; j < res.cols(); j++)

254  for (int i = 0; i < res.rows(); i++)

255  res(i,j).vi\_ = baseVari->\_variRefC[pos++];

256

257  return res;

258  }

259

267  template <int R1,int C1,int R2,int C2>

268  inline Eigen::Matrix<var,R1,C2>

269  mdivide\_left\_ldlt(const stan::math::LDLT\_factor<var,R1,C1> &A,

270  const Eigen::Matrix<double,R2,C2> &b) {

271  Eigen::Matrix<var,R1,C2> res(b.rows(),b.cols());

272

273  stan::math::check\_multiplicable("mdivide\_left\_ldlt(%1%)",A,"A",

274  b,"b",(double\*)0);

275

276  mdivide\_left\_ldlt\_vd\_vari<R1,C1,R2,C2> \*baseVari = new mdivide\_left\_ldlt\_vd\_vari<R1,C1,R2,C2>(A,b);

277

278  int pos = 0;

279  for (int j = 0; j < res.cols(); j++)

280  for (int i = 0; i < res.rows(); i++)

281  res(i,j).vi\_ = baseVari->\_variRefC[pos++];

282

283  return res;

284  }

285

293  template <int R1,int C1,int R2,int C2>

294  inline Eigen::Matrix<var,R1,C2>

295  mdivide\_left\_ldlt(const stan::math::LDLT\_factor<double,R1,C1> &A,

296  const Eigen::Matrix<var,R2,C2> &b) {

297  Eigen::Matrix<var,R1,C2> res(b.rows(),b.cols());

298

299  stan::math::check\_multiplicable("mdivide\_left\_ldlt(%1%)",A,"A",

300  b,"b",(double\*)0);

301

302  mdivide\_left\_ldlt\_dv\_vari<R1,C1,R2,C2> \*baseVari = new mdivide\_left\_ldlt\_dv\_vari<R1,C1,R2,C2>(A,b);

303

304  int pos = 0;

305  for (int j = 0; j < res.cols(); j++)

306  for (int i = 0; i < res.rows(); i++)

307  res(i,j).vi\_ = baseVari->\_variRefC[pos++];

308

309  return res;

310  }

311

312  }

313 }

314 #endif

LDLT\_alloc.hpp

check\_multiplicable.hpp

C\_

Eigen::Matrix< double, R2, C2 > C\_

**Definition:** mdivide\_left\_ldlt.hpp:24

stan::agrad::memalloc\_

memory::stack\_alloc memalloc\_

**Definition:** var\_stack.cpp:16

N\_

int N\_

**Definition:** mdivide\_left\_ldlt.hpp:41

\_alloc\_ldlt

const LDLT\_alloc< R1, C1 > \* \_alloc\_ldlt

**Definition:** mdivide\_left\_ldlt.hpp:45

\_alloc

mdivide\_left\_ldlt\_alloc< R1, C1, R2, C2 > \* \_alloc

**Definition:** mdivide\_left\_ldlt.hpp:44

stan::math::check\_multiplicable

bool check\_multiplicable(const char \*function, const T1 &y1, const char \*name1, const T2 &y2, const char \*name2, T\_result \*result)

**Definition:** check\_multiplicable.hpp:15

stan::agrad::mdivide\_left\_ldlt

Eigen::Matrix< fvar< T2 >, R1, C2 > mdivide\_left\_ldlt(const stan::math::LDLT\_factor< double, R1, C1 > &A, const Eigen::Matrix< fvar< T2 >, R2, C2 > &b)

Returns the solution of the system Ax=b given an LDLT\_factor of A.

**Definition:** mdivide\_left\_ldlt.hpp:25

stan::math::LDLT\_factor

**Definition:** LDLT\_factor.hpp:16

\_variRefC

vari \*\* \_variRefC

**Definition:** mdivide\_left\_ldlt.hpp:43

stan::math::rows

size\_t rows(const Eigen::Matrix< T, R, C > &m)

**Definition:** rows.hpp:12

var.hpp

stan::math::cols

size\_t cols(const Eigen::Matrix< T, R, C > &m)

**Definition:** cols.hpp:12

Eigen.hpp

M\_

int M\_

**Definition:** mdivide\_left\_ldlt.hpp:40

\_ldltP

boost::shared\_ptr< Eigen::LDLT< Eigen::Matrix< double, R1, C1 > > > \_ldltP

This share\_ptr is used to prevent copying the LDLT factorizations for mdivide\_left\_ldlt(ldltA,b) when ldltA is a LDLT\_factor<double>.

**Definition:** mdivide\_left\_ldlt.hpp:23

\_variRefB

vari \*\* \_variRefB

**Definition:** mdivide\_left\_ldlt.hpp:42

LDLT\_factor.hpp


---

[ Stan Home Page ]

*© 2011–2014,
Stan Development Team.*
